# Comparison of Risk-Scoring Systems in Predicting Kawasaki Disease Associated Coronary Artery Dilation in a North American Cohort

**DOI:** 10.1007/s00246-024-03611-9

**Published:** 2024-08-04

**Authors:** Elridge Schwartzenburg, Jacob Strelow, Shahryar M. Chowdhury

**Affiliations:** 1 Department of Pediatrics, Medical University of South Carolina, Charleston, SC, USA

**Keywords:** Kawasaki, Scoring systems, Pediatric cardiology, Coronary artery dilation

## Abstract

Scoring systems used to predict morbidity in children with Kawasaki disease (KD) have been developed and validated in Asian populations. The objective of this study was to assess their utility in predicting the development of coronary artery dilation in children with KD in North America. This was a secondary analysis of a National Institutes of Health / National Heart, Lung, and Blood Institute (NIH/NHLBI) Pediatric Heart Network public use dataset from a multicenter, randomized controlled trial of pulse steroid therapy in KD in a North American cohort. The primary outcome of interest was development of coronary artery dilation. The Harada, Kobayashi, Egami, and Sano scoring systems, originally developed to predict risk of intravenous immunoglobulin (IVIG) resistance in Kawasaki patients in Japan, were applied to this cohort. Subjects were kept in the analysis only if there were complete data for every element of each scoring system—Harada (n = 132), Kobayashi (n = 88), Egami (n = 139), and Sano (n = 82). Patients classified as high-risk by the Harada score were more likely to have significant coronary artery dilation (p = 0.042), were more likely to require IVIG retreatment (p = 0.002), and were more likely to require hospital readmission (p < 0.001). The Egami, Kobayashi, and Sano scores were not predictive for any measured outcome. The Harada score can be useful in identifying KD patients at risk for developing coronary artery dilation and IVIG resistance. The Harada score has demonstrated higher sensitivity but lower specificity, making it a valuable screening tool that may benefit from supplementary diagnostic methods.

## Introduction:

Kawasaki disease (KD) is a febrile illness in children involving a wide array of inflammatory symptoms including conjunctivitis, rash, mucositis, extremity changes, and/ or lymphadenopathy. KD is pathologically characterized as a vasculitis of medium-sized arteries with a predilection for coronary arteries. A well-known complication of KD is the development of coronary artery aneurysms, making KD the most common cause of acquired heart disease in children of developed countries. The current management of KD involves prompt administration of aspirin and intravenous immunoglobulin (IVIG). IVIG istypically initiated in an inpatient setting, making KD a common cause of admission to pediatric hospitals [1].

Early identification of patients at high-risk for complications of KD, specifically coronary artery dilation development, is important in guiding management and surveillance of the disease. Several scoring systems to identify high-risk KD patients have already been developed in Asia to predict IVIG resistance. However, differences in the Asian and North American populations’ genetic, environmental, and immunologic factors make the clinical utility of these scores unclear when utilized in the North American population. In addition, the utility of these scores to predict the development of coronary artery dilation is unknown. The purpose of this study was to apply these Asian scoring systems to a North American cohort and to determine their usefulness in identifying patients at high-risk for coronary artery dilation.

## Methods

We performed a secondary analysis of a NIH/NHLBI-sponsored Pediatric Heart Network public dataset produced from a multicenter, randomized, double-blind, placebo-controlled trial of pulsed corticosteroid therapy for primary treatment of KD (https://www.pediatricheartnetwork.org/studies/kawasaki-disease/). This KD trial collected data from December 2002 to December 2004 across eight centers in North America (including the US and Canada). The trial is registered with clinicaltrials.gov (NCT00132080). The eligibility criteria, methods, and results of the trial were previously published and are summarized here. [2] Eligible patients met modified American Heart Association criteria for KD [3] and were enrolled between days 4 and 10 of illness, with day 1 defined as the first day of fever. After study drug administration, all children received IVIG dosed at 2 g/kg over 10 h. They also received high dose aspirin, 80–100 mg/kg/day, until they were afebrile for 48 h followed by low dose aspirin, 3–5 mg/kg/day, until study completion. Echocardiograms and laboratory data were obtained at baseline (prior to treatment), 1 week after randomization, and 5 weeks after randomization. The results of the primary study did not provide support for the addition of a single pulsed-dose of intravenous methylprednisolone to conventional intravenous immune globulin therapy for the routine primary treatment of children with Kawasaki disease.

### Outcome Measures

The primary outcome of this study was the development of coronary artery dilation 5 weeks after randomization. Two-dimensional echocardiographic data were obtained from the public use dataset. Z-scores of the maximal internal diameters of the proximal left anterior descending and proximal right coronary artery were recorded. The larger of the Z-scores for the right coronary artery or the left anterior descending coronary artery was used to determine having a dilated coronary artery. Patients were classified as having normal (z-score < + 2.5 normalized for BSA) or dilated (z-score ≥ + 2.5) coronary arteries.

The secondary outcomes included IVIG resistance, defined as a recrudescent fever (≥ 38 °C) at least 48 h after completion of initial IVIG treatment, requiring a repeat dose or doses of IVIG. Early identification of these patients can be important since IVIG resistance has been associated with increased risk for coronary artery aneurysm development [4]. This was only assessed in the Harada score analysis as results for the other three scores have been published previously in this cohort [5]. Other secondary outcomes included need for hospital readmission and length of hospital stay.

#### *Kawasaki Disease* Scores

The Harada score was originally used to establish an indication for IVIG treatment. The Harada score assigns 1 point to each of the following: white blood cell count (WBC) > 12,000/μL, platelet count < 350,000/μL, C-reactive protein (CRP) > 3 mg/dL, hematocrit < 35%, albumin < 3.5 g/dL, age ≤ 12 months, and male sex (Table 1). A Harada score of 4 or greater has been shown to be an effective screening tool in identifying high-risk KD patients in Japanese patients [6].

The Egami scoring system was created to identify patients at risk for IVIG resistance in Japanese populations [7]. The Egami score assigns 1 point for infants < 6 months old, < 4 days of fever, platelet count ≤ 300,000 /μL, and CRP ≥ 8 mg/dL; 2 points are assigned to alanine transaminase (ALT) ≥ 80 IU/L (Table 2). A total Egami score of ≥ 3 is indicative of high-risk for IVIG resistance. The Egami score has been shown to have poor sensitivity when applied to both a US and a Mediterranean population [8, 9].

The Kobayashi scoring system was also created to identify patients at risk for IVIG resistance in Japanese populations [10]. The Kobayashi score assigns 2 points to the following: sodium ≤ 133 mmol/L, days of illness at initial treatment ≤ 4, aspartate aminotransferase (AST) ≥ 100 IU/L, % neutrophils ≥ 80%; and 1 point to the following: CRP ≥ 10 mg/dL, age ≤ 12 months, and platelet count ≤ 300,000/μL (Table 3). A total Kobayashi score ≥ 4 is indicative of high-risk for IVIG resistance.

The Sano scoring system was also created to identify patients at risk for IVIG resistance in Japanese populations [11]. The Sano score identifies three predictors of IVIG resistance including CRP ≥ 7 mg/dL, total bilirubin ≥ 0.9 mg/dL, and AST ≥ 200 IU/L (Table 4). Presence of at least two of the three indicates a high-risk Sano score.

### Statistical Methods

Differences in outcomes of interest in the high-risk vs low-risk scoring patients in each of the Harada, Egami, Kobayashi, and Sano cohort groups were assessed. Patients were included in each score group only if they had all components of that score available in the dataset (Table 5). Chi-square tests were utilized when comparing categorical variables between the high and low-risk groups. When comparing continuous variables between the two groups, independent T-tests or Mann Whitney U tests were used depending on the distribution of the data. Receiver operating curve analysis was used to determine the performance of scores of interest. A p-value of < 0.05 was considered significant. Statistical program IBM SPSS v.25 (Andover, MA) was used to perform the analysis.

## Results

A total of 190 patients were available in the public use dataset. We had complete data available to calculate a Harada score in 132/190 patients, Egami score in 139/190 patients, Kobayashi score in 88/190 patients, and Sano score in 82/190 patients. We used the baseline (prior to treatment) laboratory data to calculate all scores. The published cutoff values for each score were utilized as described above.

### Harada Score Analysis

In the 132 patients where a Harada score could be calculated, 64 out of 132 (48%) subjects were considered high-risk with a score of ≥ 4. Coronary artery dilations were found in 13 of 64 (20%) patients with high-risk Harada scores compared to only 5 of 68 (7%) patients with a low-risk Harada score (p = 0.042). A Harada score of ≥ 4 had a 72% sensitivity and 55% specificity in identifying patients at risk for coronary artery dilation development, the c-statistic was 0.68, p = 0.014. IVIG resistance with recrudescent fevers requiring IVIG retreatment was found in 17 of the 64 (27%) patients with high-risk Harada scores compared to only 4 of the 68 (6%) patients with a low-risk Harada score (p = 0.002). A Harada score of ≥ 4 had an 81% sensitivity and 58% specificity in identifying patients at risk for IVIG resistance the c-statistic was 0.71, p < 0.01. Hospital readmission was found in 17 of the 64 (27%) patients with high-risk Harada scores compared to only 3 of the 68 (4%) patients with a low-risk Harada score (p < 0.001).

The mean days of fever in the Harada cohort was not significantly different between high-risk and low-risk Harada score groups (6.5 ± 1.5 vs 6.8 ± 1.7 mean days respectively, p = 0.318). Additionally, the mean hospital length of stay had no significant difference between high-risk and low-risk Harada score groups (3.4 ± 1.7 vs 3.1 ± 1.3 mean days respectively, p = 0.223). High-risk Harada patients treated with pulse steroids had no difference in mean coronary artery diameter Z-score or any other outcome measure compared to the placebo group.

### Egami Score Analysis

In the 139 patients where an Egami score could be calculated, 29 (21%) patients were considered high-risk. Overall, there were no significant differences in any of our outcomes of interest in the high-risk Egami cohort compared to the low-risk Egami cohort. In the high-risk Egami cohort, 5/29 (17%) patients had coronary artery abnormalities compared to 13/110 (12%) patients with low-risk Egami scores, p = 0.533. In the high-risk Egami cohort, 5 of the 29 (17%) patients required hospital readmission compared to 15 of the 110 (14%) patients with low-risk Egami scores (p = 0.766). Length of stay was no different in the high-risk or low-risk groups.

### Kobayashi Score Analysis

In the 88 patients where a Kobayashi score could be calculated, 7 of the 88 (8%) had a high-risk Kobayashi score. In the high-risk Kobayashi cohort, 2 of 7 (29%) patients had coronary artery abnormalities compared to 9 of 81 (11%) patients with low-risk Kobayashi scores, p = 0.210. In the high-risk Kobayashi cohort, 1 of 7 (14%) patients required hospital readmission compared to 10 of 81 (12%) patients with low-risk Kobayashi scores, p = 1.000. The mean hospital length of stay in our high-risk vs low-risk Kobayashi cohorts were 5.1 ± 2.7 and 3.0 ± 0.7 days, respectively (p < 0.001).

### Sano Score Analysis

In the 82 patients where a Sano score could be calculated, 16 of the 82 (20%) had high-risk Sano scores. In the high-risk Sano cohort, 3 of 16 (19%) patients had coronary artery abnormalities compared to 7 of 66 (11%) patients with low-risk Sano scores, p = 0.401. In the high-risk Sano cohort, 2 of 16 (13%) patients required hospital readmission compared to 11 of the 66 (17%) patients with low-risk Sano scores, p = 0.735. Length of stay was no different in the high-risk or low-risk groups.

## Discussion

To our knowledge, this is the first study to compare multiple Kawasaki scoring systems’ ability to risk stratify patients at high-risk for coronary artery dilation, in addition to other outcomes, in a large multicenter North American cohort. The main finding of this study was that the Harada score was the only score investigated that can be useful in identifying patients at high-risk for coronary artery dilation, IVIG resistance, and hospital readmission. This study supports the utility of the Harada score in a broad North American KD population. This study’s findings are supported by two previous single-center studies in North America showing the Harada score to be useful in predicting the development of coronary artery dilation [12, 13]. The Harada score has also shown similar discriminatory ability in detecting IVIG resistance to the other scores in Asia [14].

The Harada score is clinically feasible to obtain in the majority of KD patients in the current era. The components of the score are routinely available in nearly all patients at the time of initial diagnosis of KD.

The sensitivity and specificity for the Harada score in predicting coronary artery dilation (72% and 55%, respectively) and IVIG resistance (81% and 58%, respectively) make for a valuable screening tool. But with weaker specificity, clinicians may benefit from supplemental diagnostic methods to confirm high-risk cases. Additionally, the c-statistic values of 0.68 for coronary artery dilation risk and 0.71 for IVIG resistance risk indicate the Harada score’s poor to acceptable discrimination ability highlighting additional limitations and the necessity for ongoing validation and adjustments.

In the context of reasonable sensitivity in predicting IVIG resistance and a positive correlation to hospital readmission, a Harada score could influence timing of hospital discharge and outpatient follow-up. Considering the sensitivity in predicting coronary artery dilation, a Harada score could also impact timing of follow-up imaging by echocardiogram and if there is a need for advanced coronary imaging. Our study supports the usefulness of the Harada score as a supplemental clinical tool in individual patient care but should not replace the current standard of care for KD diagnosis and management.

### Limitations

This study allowed different sample sizes in each scoring cohort. This may bias the results to have positive results in the larger cohorts such as the Harada cohort with 132 patients with complete data to be included in analysis compared to Kobayashi and Sano cohorts which only had 88 and 82 patients respectively with complete data. Baseline coronary artery Z-score > 2.0 has been used to identify patients at risk for developing coronary artery dilation [12]. We chose not to include this criterion in the analysis because clinically any patient with a baseline Z-score > 2.0 is treated as high-risk and receives close follow-up. This study could not assess the usefulness of these scores in predicting outcomes in incomplete Kawasaki disease as this data was not included in the public use dataset.

## Conclusion

The Harada score can be useful in identifying KD patients at risk for developing significant coronary artery dilation, IVIG resistance, and hospital readmission. The Harada score has demonstrated higher sensitivity but lower specificity, making it a valuable screening tool that may benefit from supplementary diagnostic methods to confirm high-risk cases. The Harada score’s ability to predict high-risk outcomes in a North American KD cohort underscores its potential utility, but also highlights the necessity for ongoing validation and adjustment to enhance its accuracy and reliability.

## Figures and Tables

**Table 1:** Harada score

Variable	Cutoff	Score

WBC	> 12,000/μL	1
Platelets	< 350,000/μL	1
CRP	> 3 mg/dL	1
Hematocrit	< 35%	1
Albumin	< 3.5 g/dL	1
Age	≤ 12 months	1
Sex	Male	1

Low Risk: 0–3

High Risk: ≥ 4

**Table 2:** Egami score

Variable	Cutoff	Score

Age	< 6 months	1
Fever	< 4 days	1
Platelets	≤ 300,000/ μL	1
CRP	≥ 8 mg/dL	1
ALT	≥ 80 IU/L	2

Low Risk: 0–2

High Risk: ≥ 3

**Table 3:** Kobayashi score

Variable	Cutoff	Score

Sodium	≤ 133 mmol/L	2
Fever	≤ 4 days	2
AST	≥ 100 IU/L	2
%Neutrophils	≥ 80%	2
CRP	≥ 10 mg/dL	1
Age	≤ 12 months	1
Platelets	≤ 300,000/μL	1

Low Risk: 0–3

High Risk: ≥ 4

**Table 4:** Sano score

Variable	Cutoff	Score

CRP	≥ 7 mg/dL	1
Total bilirubin	≥ 0.9 mg/dL	1
AST	≥ 200 IU/L	1

Low Risk: 0–1

High Risk: ≥ 2

**Table 5:** Demographic, laboratory, and outcome data

	Harada score group (n = 132)	Egami score group (n = 139)	Kobayashi score group (n = 88)	Sano score group (n = 82)

Age (years)	3.1 (1.5, 4.8)	3.1 (1.5, 4.6)	3.2 (1.5, 4.6)	3.2 (1.7, 4.5)
Male Sex, n (%)	84 (64%)	87 (63%)	54 (61%)	54 (66%)
Race, n (%)				
White	80 (61%)	83 (60%)	52 (59%)	50 (61%)
Black	25 (19%)	27 (19%)	18 (21%)	16 (19%)
Asian	15 (11%)	16 (12%)	11 (13%)	8 (10%)
Other	12 (9%)	13 (9%)	7 (8%)	8 (10%)
Hispanic	23 (17%)	24 (17%)	18 (21%)	15 (18%)
WBC (× 10^−3^/mm^3^)	13.0 (10.0, 16.7)	13.0 (10.0, 16.6)	13.3 (10.0, 18.0)	13.2 (10.0, 17.0)
Hemoglobin (g/dL)	10.9 (10.0, 11.7)	10.9 (10.1, 11.7)	10.8 (9.9, 11.6)	10.8 (9.8, 11.6)
Hematocrit	32.0 (29.3, 34.0)	32.0 (30.0, 34.0)	32.0 (29.0, 34.0)	32.0 (29.0, 34.0)
Platelet (× 10^−3^/mm^3^)	388 (309, 463)	389 (301, 466)	383 (303, 463)	394 (319, 465)
C-reactive protein (mg/dL)	8.4 (4.2, 17.2)	8.3 (4.2, 16.8)	10.3 (5.4, 19.3)	10.0 (4.3, 19.0)
ESR (mm/hr)	65 (43, 91)	66 (42, 91)	70 (51, 98)	65 (42, 100)
Sodium (mmol/L)	136 (134, 138)	136 (134, 138)	136 (134, 139)	136 (134, 138)
AST (units/L)	35 (26, 56)	35 (26, 56)	35 (26, 56)	38 (27, 60)
ALT (units/L)	36 (16, 83)	36 (16, 83)	38 (16, 84)	46 (17, 93)
Albumin (g/dL)	3.3 (2.8, 3.7)	3.3 (2.8, 3.7)	3.4 (2.8, 3.7)	3.2 (2.7, 3.7)
Bilirubin (mg/dL)	0.3 (0.2, 0.8)	0.3 (0.2, 0.9)	0.3 (0.2, 0.9)	0.3 (0.2, 0.9)
IgG (mg/dL)	684 (512, 865)	684 (511, 868)	680 (499, 875)	689 (534, 864)
IgM (mg/dL)	109 (68, 141)	109 (69, 140)	110 (68, 144)	109 (71, 139)
IgA (mg/dL)	89 (55, 130)	86 (54, 129)	86 (60, 135)	90 (60, 128)
Steroid Treatment, n (%)	60 (46%)	62 (45%)	35 (40%)	36 (44%)
Fever days, n (%)	6 (6, 8)	6 (6, 8)	6 (6, 8)	6 (6, 8)
Baseline proximal LAD Z-score	0.42 (−0.44, 1.29)	0.48 (−0.42, 1.26)	0.36 (−0.47, 1.24)	0.54 (−0.26, 1.33)
Baseline proximal RCA Z-score	0.79 (0.05, 1.55)	0.74 (0.05, 1.51)	0.80 (0.15, 1.61)	0.73 (0.24, 1.54)
Baseline maximum coronary Z-score	1.13 (0.33, 1.76)	1.11 (0.34, 1.75)	0.99 (0.40, 1.76)	1.12 (0.39, 1.80)
Retreatment, n (%)	21 (16%)	21 (15%)	12 (14%)	13 (16%)
Length of Stay (days)	3 (3, 3)	3 (3, 3)	3 (3, 3)	3 (3, 3)

Results are reported in median (interquartile range) or count (percentage)

*ESR* Erythrocyte sedimentation rate, *LAD* left anterior descending, *RCA* right coronary artery

## Data Availability

We performed a secondary analysis of a NIH/NHLBI-sponsored Pediatric Heart Network public dataset produced from a multicenter, randomized, double-blind, placebo-controlled trial of pulsed corticosteroid therapy for primary treatment of KD (https://www.pediatricheartnetwork.org/studies/kawasaki-disease/). The trial is registered with clinicaltrials.gov (NCT00132080).
